# Generation of KCL012 research grade human embryonic stem cell line carrying a mutation in the HTT gene

**DOI:** 10.1016/j.scr.2016.01.012

**Published:** 2016-03

**Authors:** Laureen Jacquet, Heema Hewitson, Victoria Wood, Neli Kadeva, Glenda Cornwell, Stefano Codognotto, Carl Hobbs, Emma Stephenson, Dusko Ilic

**Affiliations:** a Stem Cell Laboratories, Division of Women's Health, Faculty of Life Sciences and Medicine, King's College London and Assisted Conception Unit, Guys' Hospital, London, United Kingdom; b Histology Laboratory, Wolfson Centre for Age-Related Diseases, Faculty of Life Sciences and Medicine, King's College London, United Kingdom

## Abstract

The KCL012 human embryonic stem cell line was derived from an embryo donated for research that carried an autosomal dominant mutation affecting one allele of the HTT gene encoding huntingtin (46 trinucleotide repeats; 17 for the normal allele). The ICM was isolated using laser microsurgery and plated on γ-irradiated human foreskin fibroblasts. Both the derivation and cell line propagation were performed in an animal product-free environment. Pluripotent state and differentiation potential were confirmed by in vitro and in vivo assays.

## Resource table

**Table:** 

Name of stem cell line	KCL012
Institution	King's College London, London UK
Derivation team	Neli Kadeva, Victoria Wood, Glenda Cornwell, Stefano Codognotto, Emma Stephenson
Contact person and email	Dusko Ilic, email: dusko.ilic@kcl.ac.uk
Type of resource	Biological reagent: cell line
Sub-type	Human pluripotent stem cell line
Origin	Human embryo
Key marker expression	Pluripotent stem cell markers: NANOG, OCT4, TRA-1-60, TRA-1-81, alkaline phosphatase (AP) activity
Authentication	Identity and purity of line confirmed
Link to related literature (direct URL links and full references)	1) Ilic, D., Stephenson, E., Wood, V., Jacquet, L., Stevenson, D., Petrova, A., Kadeva, N., Codognotto, S., Patel, H., Semple, M., Cornwell, G., Ogilvie, C., Braude, P., 2012. Derivation and feeder-free propagation of human embryonic stem cells under xeno-free conditions. Cytotherapy. 14 (1), 122–128. doi: 10.3109/14653249.2011.623692 http://www.ncbi.nlm.nih.gov/pubmed/22029654 2) Stephenson, E., Jacquet, L., Miere, C., Wood, V., Kadeva, N., Cornwell, G., Codognotto, S., Dajani, Y., Braude, P., Ilic, D., 2012. Derivation and propagation of human embryonic stem cell lines from frozen embryos in an animal product-free environment. Nat. Protoc. 7 (7), 1366–1381. doi: 10.1038/nprot.2012.080 http://www.ncbi.nlm.nih.gov/pubmed/22722371
Information in public databases	KCL012 is a National Institutes of Health (NIH) registered hESC line NIH Registration Number: 0213 NIH Approval Number: NIHhESC-13-0213 http://grants.nih.gov/stem_cells/registry/current.htm?id=650
Ethics	The hESC line KCL012 is derived under license from the UK Human Fertilisation and Embryology Authority (research license numbers: R0075 and R0133) and also has local ethical approval (UK National Health Service Research Ethics Committee Reference: 06/Q0702/90). Informed consent was obtained from all subjects and the experiments conformed to the principles set out in the WMA Declaration of Helsinki and the NIH Belmont Report. No financial inducements are offered for donation.

## Resource details

**Table:** 

Consent signed	Aug. 12, 2009
Embryo used	Aug. 23, 2009
UK Stem Cell Bank Deposit Approval	Sep. 23, 2010 Reference: SCSC10-32
Sex	Male 46, XY
Grade	Research
Disease status (Fig. 1)	Mutation affecting one allele of the HTT gene encoding huntingtin (~ 46 CAG repeats; 17 for the normal allele) associated with Huntington's disease (Ilic et al., 2012)
Karyotype (G banding) (Fig. 2)	No imbalance detected
Karyotype (aCGH)	No imbalance detected
DNA fingerprint	Allele sizes (in bp) of 17 microsatellite markers specific for chromosomes 13, 18 and 21 (Ilic et al., 2012)
Viability testing	Pass
Pluripotent markers (immunostaining) (Fig. 3)	NANOG, OCT4, TRA-1-60, TRA-1-81, AP activity (Ilic et al., 2012)
Three germ layers differentiation in vitro (immunostaining) (Fig. 4)	Endoderm: AFP (α-fetoprotein); Ectoderm: TUBB3 (tubulin, β3 class III); Mesoderm: ACTA2 (actin, α2, smooth muscle) (Ilic et al., 2012)
Three germ layer differentiation in vivo (teratomas) (Fig. 5)	Endoderm: AFP, GATA4 Ectoderm: TUBB3, GFAP (glial fibrillary acidic protein) Mesoderm: DES (desmin), Alcian Blue and periodic acid–Schiff (PAS)-stained cartilage
Sibling lines available	KCL013

**Fig. 3:**
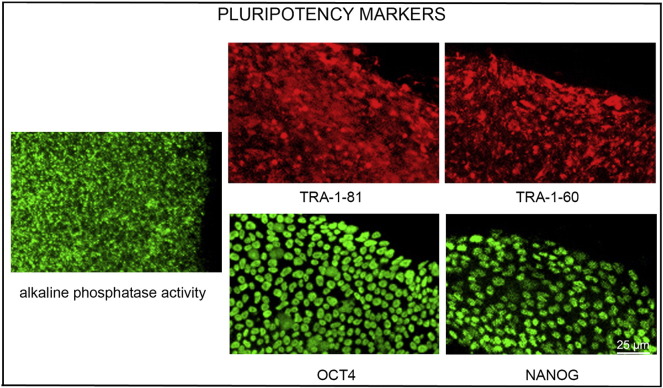
Expression of pluripotency markers. Pluripotency is confirmed by immunostaining (Oct4, Nanog, TRA-1-60, TRA-1-81) and alkaline phosphatase (AP) activity assay. Scale bar, 25 μm.

**Fig. 4:**
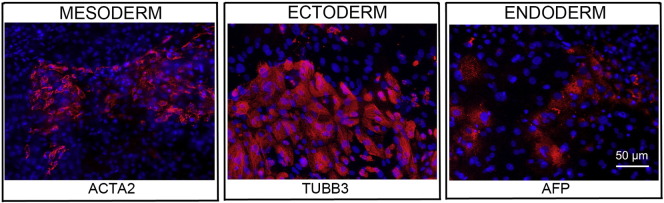
Differentiation of three germ layers in vitro is confirmed by detection of markers: smooth muscle actin (ACTA2, red) for mesoderm, β-III tubulin (TUBB3, red) for ectoderm and α-fetoprotein (AFP, red) for endoderm. Nuclei are visualized with Hoechst 33342 (blue). Scale bar, 50 μm.

**Fig. 5:**
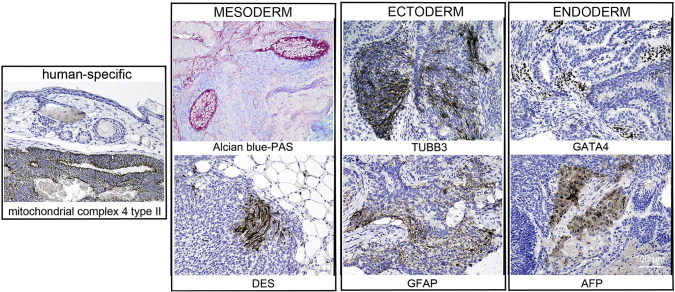
Differentiation of three germ layers in vivo. Teratomas were encapsulated and did not invade surrounding tissue. Sections are counterstained with hematoxylin and eosin and specific stains are brown (immunohistochemistry) or light blue (Alcian blue). Germ layer markers: Alcian blue–PAS-stained cartilage and DES for mesoderm, TUBB3 and GFAP for ectoderm, GATA4 and AFP for endoderm. Positive immunostaining for complex IV type II marker confirms the human origin of the tumor. Scale bars are 100 μm.

We generated KCL012 clinical grade hESC line following protocols, established previously (Ilic et al., 2012, Stephenson et al., 2012). The expression of the pluripotency markers was tested after freeze/thaw cycle (Fig. 3; Ilic et al., 2012). Differentiation potential into three germ layers was verified in vitro (Fig. 4; Ilic et al., 2012) and in vivo (Fig. 5).

## Materials and methods

### Consenting process

We distribute Patient Information Sheet (PIS) and consent form to the in vitro fertilization (IVF) patients if they opted to donate to research embryos that were stored for 5 or 10 years. They mail signed consent back to us and that might be months after the PIS and consent were mailed to them. If in the meantime new versions of PIS/consent are implemented, we do not send these to the patients or ask them to re-sign; the whole process is done with the version that was given them initially. The PIS/consent documents (PGD-V.6) were created on Aug. 10, 2007. HFEA Code of Practice that was in effect at the time of document creation: Edition 7 – R.1 (http://www.hfea.gov.uk/2999.html). The donor couple signed the consent on Oct. 15, 2009. HFEA Code of Practice that was in effect at the time of donor signature: Edition 8 – R.1. HFEA Code of Practice Edition 7 – R.1 was in effect until Dec. 09, 2007 and Edition 8 – R.1 was in effect: Oct. 01, 2009–Apr. 06, 2010.

### Embryo culture and micromanipulation

Embryo culture and laser-assisted dissection of inner cell mass (ICM) were carried out as previously described in details (Ilic et al., 2012, Stephenson et al., 2012). The cellular area containing the ICM was then washed and transferred to plates containing mitotically inactivated human neonatal foreskin fibroblasts (HFF).

### Cell culture

ICM plated on mitotically inactivated HFF was cultured as described (Ilic et al., 2012, Stephenson et al., 2012). Trophectoderm cells were removed mechanically from outgrowth (Ilic et al., 2007, Ilic et al., 2010). hESC colonies were expanded and cryopreserved at the third passage.

### Viability test

Straws with the earliest frozen passage (p.2–3) are thawed and new colonies are counted three days later. These colonies are then expanded up to passage 8, at which point cells were part frozen and part subjected to standard battery of tests (pluripotency markers, in vitro and in vivo differentiation capability, genetics, sterility, mycoplasma).

### Pluripotency markers

Pluripotency was assessed using two different techniques: enzymatic activity assay [alkaline phosphatase (AP) assay] and immunostaining as described (Fig. 3; Ilic et al., 2012, Stephenson et al., 2012).

### Genotyping

DNA was extracted from hESC cultures using a Chemagen DNA extraction robot according to the manufacturer's instructions. Amplification of polymorphic microsatellite markers was carried out as described (Ilic et al., 2012). Allele sizes were recorded to give a unique fingerprint of each cell line.

### Differentiation

Spontaneous differentiation into three germ layers was assessed in vitro and in vivo as described (Petrova et al., 2014, Stephenson et al., 2012).

### Array comparative genomic hybridization (aCGH)

aCGH was performed as described in details (Ilic et al., 2012).

## Author disclosure statement

There are no competing financial interests in this study.

## Figures and Tables

**Fig. 1:**
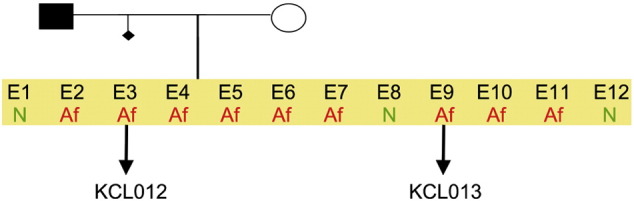
Genetic pedigree tree. The couple undergoing IVF had 12 embryos in this particular cycle. Three embryos were normal, whereas nine carried the mutation in HTT and were donated for research. We derived hESC lines from two of them.

**Fig. 2:**
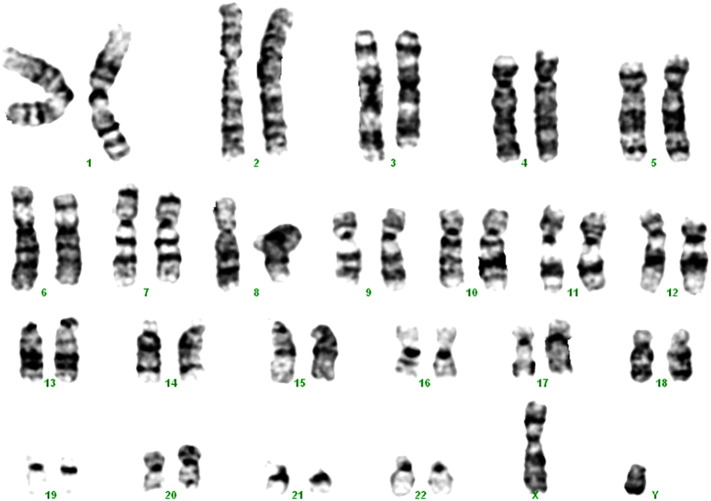
A modal karyotype (in 18 cells) showed a normal male chromosome complement and banding pattern. In addition, two anomalous cells were seen, believed to be the result of harvesting artifact: one cell 45,XY,-17; one cell 45,XY,-21.
